# A single-center, retrospective study of COVID-19 features in children: a descriptive investigation

**DOI:** 10.1186/s12916-020-01596-9

**Published:** 2020-05-06

**Authors:** Huijing Ma, Jiani Hu, Jie Tian, Xi Zhou, Hui Li, Maxwell Thomas Laws, Luke David Wesemann, Baiqi Zhu, Wei Chen, Rafael Ramos, Jun Xia, Jianbo Shao

**Affiliations:** 1 Imaging Center, Wuhan Children’s Hospital (Wuhan Maternal and Child Healthcare Hospital), Tongji Medical College, Huazhong University of Science & Technology, No.100 Hongkong Road, Wuhan, 430016 China; 2 Department of Radiology, School of Medicine, Wayne State University, Detroit, MI 48201 USA; 3 Beijing Advanced Innovation Center for Big Data-Based Precision Medicine, School of Medicine and Engineering, Beihang University, Beijing, 100191 China; 4 Department of Radiology, Shenzhen Second People’s Hospital, The First Affiliated Hospital of Shenzhen University Health Science Center, 3002 SunGang Xi Road West, Shenzhen, 518035 China; 5 Medical department, Wuhan Children’s Hospital (Wuhan Maternal and Child Healthcare Hospital), Tongji Medical College, Huazhong University of Science & Technology, No.100 Hongkong Road, Wuhan, 430016 China; 6 Department of Radiology, Tongji Hospital, Tongji University School of Medicine, Shanghai, 200065 China; 7 Pingshan District People’s Hospital, Pingshan General Hospital of Southern Medical University, Shenzhen, 518118 Guangdong China

**Keywords:** Children, Pediatric, Coronavirus, COVID-19, SARS-CoV-2, Epidemiology, Clinical features, Computerized tomography

## Abstract

**Background:**

Compared to adults, there are relatively few studies on COVID-19 infection in children, and even less focusing on the unique features of COVID-19 in children in terms of laboratory findings, locations of computerized tomography (CT) lesions, and the role of CT in evaluating clinical recovery. The objective of this study is to report the results from patients at Wuhan Children’s Hospital, located within the initial center of the outbreak.

**Methods:**

Clinical, imaging, and laboratory data of 76 children were collected retrospectively and analyzed with the Fisher exact test and Cox regression statistical methods.

**Results:**

Among 50 children with a positive COVID-19 real-time reverse-transcriptase polymerase chain reaction (PCR), five had negative PCR results initially but showed positive results in subsequent tests. Eight (16%) patients had lymphopenia, seven (14%) with thrombocytopenia, four (8%) with lymphocytosis, two (4%) with thrombocytosis, ten (20%) with elevated C-reactive protein, four (8%) with hemoglobin above, and six (12%) with below standard reference values. Seven (14%) of the 50 had no radiologic evidence of disease on chest CT. For the 43 patients who had abnormal CT findings, in addition to previously reported patterns of ground-glass opacity (67%), local patchy shadowing (37%), local bilateral patchy shadowing (21%), and lesion location of lower lobes (65%), other CT features include that an overwhelming number of pediatric patients had lesions in the subpleural area (95%) and 22 of the 28 lower lobe lesions were in the posterior segment (78%). Lesions in most of the 15 patients (67%) who received chest CT at discharge were not completely absorbed, and 26% of these pediatric patients had CT lesions that were either unchanged or worse.

**Conclusions:**

There were a few differences between COVID-19 children and COVID-19 adults in terms of laboratory findings and CT characteristics. CT is a powerful tool to detect and characterize COVID-19 pneumonia but has little utility in evaluating clinical recovery for children. These results oppose current COVID-19 hospital discharge criteria in China, as one requirement is that pulmonary imaging must show significant lesion absorption prior to discharge. These differences between pediatric and adult cases of COVID-19 may necessitate pediatric-specific discharge criteria.

## Background

Since initially identified in Wuhan city of China’s Hubei province in December 2019, the coronavirus disease 2019 (COVID-19) has resulted in 466,836 confirmed cases and 21,152 deaths as of March 25, 2020. Two months prior, on January 23, 2020, there were only 581 reported cases. COVID-19 can rapidly spread from human-to-human and is more contagious than other notable members of the coronavirus family, such as severe acute respiratory syndrome (SARS) and Middle Eastern respiratory syndrome (MERS) [1, 2]. The World Health Organization recently declared COVID-19 a global pandemic, and the USA has declared a national emergency. Even though the incidence of COVID-19 infection in children is less than it is in adults, the total number of pediatric cases is expected to increase rapidly in the coming weeks.

Compared to adults [3–7], there are a few studies on the COVID-19 in children. Although mortality in children has been reported [8], studies have demonstrated that COVID-19 is generally less severe compared to adults in terms of both symptoms and computerized tomography (CT) manifestations [9–18]. The common chest CT patterns are ground-glass opacities (GGO) followed by local bilateral shadowing (LPS), in contrast to a large percentage of bilateral patchy shadowing (BPS) pattern in adults [19, 20]. However, there are no studies that quantitatively examine the location of lung lesions in COVID-19-positive pediatric patients [21]. Most of the pediatric patients are at the early stages of the disease when admitted to hospitals. Thus, a detailed localization study is meaningful both clinically and scientifically, as it could help pinpoint lung regions that are particularly susceptible to COVID-19 infection.

Several studies have reported on the laboratory findings of children infected with COVID-19. However, the interpretations of these results vary substantially [15, 22–24]. The discrepancy in laboratory interpretations could be attributed to the studies each referring to a different set of reference values. Of note, the range of normal lab values changes depending upon the age of the child, i.e., a 1-year-old has a different set of reference values than a 9-year-old. Confounding these results is the fact that the reference values used among the studies lack consistency and appear to be hospital-self-defined values [15, 22–24]. This inconsistency of reference values makes any systemic review of the published data less meaningful [23].

There is also no research on the role of CT in monitoring clinical recovery in children. CT has been widely used in the clinical management of adult patients due to its ability to reveal detailed features of pneumonia [25–28]. Because of how many unknowns there were about the disease, particularly at the beginning of the COVID-19 outbreak, CT was frequently used in the clinical management and diagnosis of children in China. Notably, repeated use of CT can be harmful, particularly for children [29, 30].

The objective of this study is to report relevant findings from the COVID-19-positive patients treated at Wuhan Children’s Hospital. Specifically, we attempt to answer three questions based on the patient’s clinical, laboratory, diagnostic, and treatment outcome data. The questions are, in hospitalized COVID-19 children, (i) what are the typical laboratory findings, (ii) is there any unique CT feature, and (iii) is CT necessary for evaluating clinical recovery?

## Methods

### Study design and patient selection

**Fig. 1:**
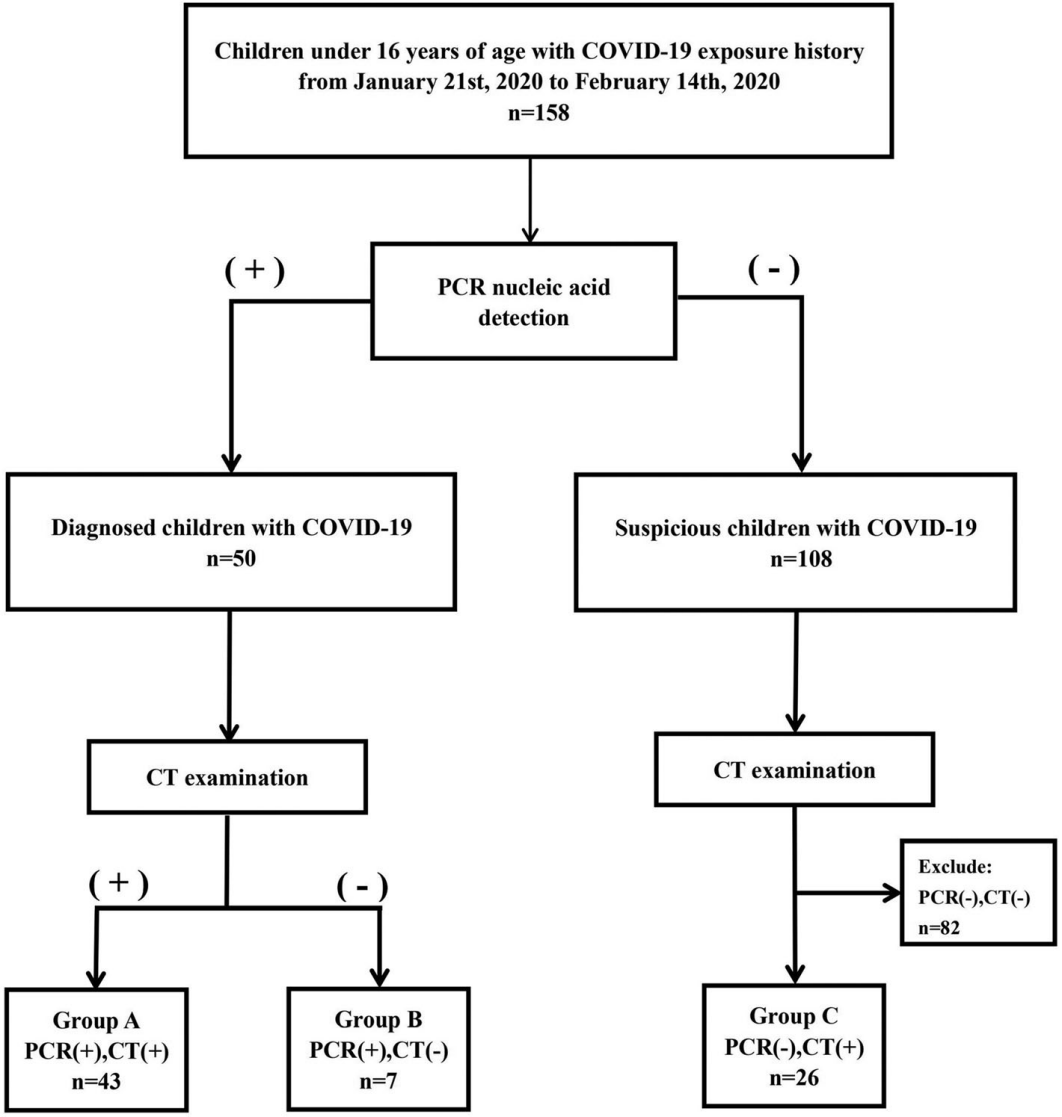
Flow chart for patient selection. Group A: 43 children with COVID-19 exposure history, positive CT, and positive PCR. Group B: seven children with COVID-19 exposure history, negative CT, and positive PCR. Group C: 26 children with COVID-19 exposure history, positive CT, and persistently negative PCR results

For this retrospective, single-center study, patients were recruited from January 21 to February 14, 2020, at Wuhan Children’s Hospital in Wuhan, China. Real-time reverse-transcriptase polymerase chain reaction (PCR) was performed on children 16 years of age and under who had a family or social history of COVID-19 exposure. Subsequently, these patients received a chest CT examination to evaluate lung pathology. Based on the PCR and CT results, these patients were stratified into groups A–C (Fig. 1). This study was approved by the Ethics Committee of Wuhan Children’s Hospital (Wuhan Maternal and Child Health Care Hospital # WHCH 2020005). Written informed parental/guardian consent and child assent (where appropriate) were obtained prior to enrollment in the study.

### Procedures

We obtained demographic information, clinical symptoms, laboratory results, management, and outcome data from each patient’s electronic medical records. Clinical outcomes were followed up to February 17, 2020.

Chest CT without intravenous contrast was performed on all patients using a Siemens SOMATOM Definition AS128 or GE Optima CT 660 with a 1-mm or 0.625-mm slice thickness, respectively. Children under 5 years old, as well as uncooperative children, received oral chloral hydrate sedation (0.5 ml/kg) prior to CT. Cooperative children above 5 years old were trained with breathing exercises prior to CT.

All CT images were reviewed by at least two radiologists with more than 10 years of experience. Imaging was reviewed independently. When the opinions on the CT features were inconsistent, the two radiologists discussed and decided together. Only final decisions reached by consensus are reported. No negative control cases were examined.

PCR confirmation of COVID-19 was performed at two different institutions: Hubei Center for Disease Control and Prevention and Wuhan Children’s Hospital.

### Patient discharge

Criteria for discharging pediatric patients in this hospital were normal body temperature for 3 days, two negative PCR results at 24-h intervals, and resolution of all clinical symptoms.

### Statistical analysis

The Fisher exact test method was used to determine whether there is a significant difference in CT image characteristics and lesion locations between group A and group C. The Cox regression analysis was used to determine whether changes in CT images during treatment were associated with clinical outcomes for children with COVID-19 infection. All analyses were performed using EmpowerStats (http://www.empowerstats.com) and the statistical package R (version 3.2.3). *p* value of less than 0.05 was considered to indicate a statistically significant difference.

## Results

From January 21 to February 14, 2020, 158 children at Wuhan Children’s Hospital were radiologically examined with chest CT, and respiratory secretions were obtained and subsequently tested for COVID-19 with PCR. A CT scan was considered positive when at least one lesion was identified. Among them, 43 had a positive CT and positive PCR (group A), 7 had a negative CT and positive PCR (group B), and 26 had a positive CT and at least two negative consecutive PCR results (group C, Fig. 1).

PCR-positive groups A and B (*n* = 50) were chosen to interpret clinical and chest CT features because group C patients were not deemed COVID-19 positive by PCR. Over half of the patients were males (56%, Table 1). The most common symptoms at the onset of illness (Table 1) were fever (64%) and cough (44%); less common symptoms were rhinorrhea (16%), abdominal pain (4%), diarrhea (6%), fatigue (4%), and pharyngalgia (2%). Six children (12%) were asymptomatic. After treatment, 38 (76%) children were discharged.

**Table 1:** Demographics and characteristics of patients

Characteristics	Group A	Group B	Group A + B	Group C
**Age, years**	3.0 (0.9–7.5)	1.0 (1.0–4.5)	2.5 (0.9–7.0)	2.5 (1.2–9.8)
> 2.5	22/43 (51%)	3/7 (43%)	25/50 (50%)	13/26 (50%)
≤ 2.5	21/43 (49%)	4/7 (57%)	25/50 (50%)	13/26 (50%)
**Sex**				
Male	23/43 (53%)	5/7 (71%)	28/50 (56%)	14/26 (54%)
Female	20/43 (47%)	2/7 (29%)	22/50 (44%)	12/26 (46%)
**Type of care**				
Discharged patients	33/43 (77%)	5/7 (71%)	38/50 (76%)	26/26 (100%)
Hospitalization duration	11.0 (9.0~13.0)	13.0 (7.0~14.0)	11.0 (8.2~13.8)	10.5 (8.0~12.0)
**Clinical status**				
Asymptomatic^a^	0/43 (0%)	2/7 (29%)	2/50 (4%)	0/26 (0%)
Very mild	0/43 (0%)	5/7 (71%)	5/50 (10%)	0/26 (0%)
Mild	41/43 (95%)	0/7 (0%)	41/50 (82%)	26/26 (100%)
Severe	0/43 (0%)	0/7 (0%)	0/50 (0%)	0/26 (0%)
Critically ill	2/43 (5%)	0/7 (0%)	2/50 (4%)	0/26 (0%)
**Symptoms**				
Fever	29/43 (67%)	3/7 (43%)	32/50 (64%)	21/26 (81%)
Cough	21/43 (49%)	1/7 (14%)	22/50 (44%)	19/26 (73%)
Myalgia or fatigue	2/43 (5%)	0/7 (0%)	2/50 (4%)	0/26 (0%)
Sore throat (pharyngalgia)	1/43 (2%)	0/7 (0%)	1/50 (2%)	0/26 (0%)
Diarrhea	3/43 (7%)	0/7 (0%)	3/50 (6%)	0/26 (0%)
Abdominal pain	2/43 (5%)	0/7 (0%)	2/50 (4%)	0/26 (0%)
Rhinorrhea	7/43 (16%)	1/7 (14%)	8/50 (16%)	2/26 (8%)
Loss of appetite	2/43 (5%)	0/7 (0%)	2/50 (4%)	0/26 (0%)
Chest pain	0/43 (0%)	0/7 (0%)	0/50(0%)	1/26 (4%)
Intussusception	1/43 (2%)	0/7 (0%)	1/50 (2%)	0/26 (0%)
No symptoms	4/43 (9%)	2/7 (29%)	6/50 (12%)	0/26 (0%)
Mortality	0/43 (0%)	0/7 (0%)	0/50 (0%)	0/26 (0%)
**Comorbidity**				
Cardiac damage	4/43 (9%)	1/7 (14%)	5/50 (10%)	3/26 (12%)
Appendicitis	1/43 (2%)	0/7 (0%)	1/50 (2%)	0/26 (0%)
Foreign body in bronchus	1/43 (2%)	0/7 (0%)	1/50 (2%)	0/26 (0%)
Mycoplasma infection	5/43 (12%)	0/7 (0%)	5/50 (10%)	11/26 (42%)
Respiratory syncytial virus infection	1/43 (2%)	0/7 (0%)	1/50 (2%)	0/26 (0%)
Renal failure	1/43 (2%)	0/7 (0%)	1/50 (2%)	0/26 (0%)
Intestinal necrosis in MODS	1/43 (2%)	0/7 (0%)	1/50 (2%)	0/26 (0%)

^a^No clinical symptoms and no abnormal CT findings

Laboratory reference normal ranges were age- and gender-adjusted according to values in *Reference Range Values for Pediatric Care 2nd edition*, pages 92–98 [31]. On laboratory assessment, eight (16%) and seven (14%) patients had lymphopenia and thrombocytopenia, respectively. In contrast, four (8%) were noted to have lymphocytosis, and two (4%) had thrombocytosis. Overall, leukopenia was observed in 19 (38%) patients and elevated C-reactive protein in ten (20%) patients. A small set of patients had hemoglobin abnormalities, four (8%) with elevated hemoglobin, and six (12%) with anemia (Table 2).

**Table 2:** Laboratory examination and CT radiographic characteristics

Characteristics	Group A	Group B	Group A + B	Group C
**Laboratory examination**^a^				
Hemoglobin count increase (↑)	2/43 (5%)	2/7 (29%)	4/50 (8%)	6/26 (23%)
Hemoglobin count normal	36/43 (83%)	4/7 (57%)	40/50 (80%)	18/26 (69%)
Hemoglobin count decrease (↓)	5/43 (12%)	1/7 (14%)	6/50 (12%)	2/26 (8%)
C-reactive protein level increase (↑)	10/43 (23%)	0/7 (0%)	10/50 (20%)	8/26 (31%)
C-reactive protein level normal	33/43 (77%)	7/7 (100%)	40/50 (80%)	18/26 (69%)
Platelet count increase (↑)	1/43 (2%)	1/7 (14%)	2/50 (4%)	1/26 (4%)
Platelet count normal	35/43 (82%)	6/7 (86%)	41/50 (82%)	18/26 (69%)
Platelet count decrease (↓)	7/43 (16%)	0/7 (0%)	7/50 (14%)	7/26 (27%)
Lymphocyte count increase (↑)	2/43 (5%)	2/7 (29%)	4/50 (8%)	2/26 (8%)
Lymphocyte count normal	34/43(79%)	4/7 (57%)	38/50 (76%)	18/26 (69%)
Lymphocyte count decrease (↓)	7/43 (16%)	1/7 (14%)	8/50 (16%)	6/26 (23%)
Blood leukocyte count increase (↑)	2/43 (5%)	0/7 (0%)	2/50 (4%)	1/26 (4%)
Blood leukocyte count normal	23/43 (53%)	6/7 (86%)	29/50 (58%)	13/26 (50%)
Blood leukocyte count decrease (↓)	18/43 (42%)	1/7 (14%)	19/50 (38%)	12/26 (46%)
**CT radiographic characteristics**				
Overall patients with CT abnormalities	43/43 (100%)	0/7 (0%)	43/50 (86%)	26/26 (100%)
Ground-glass opacity	29/43 (67%)	0/7 (0%)	29/50 (58%)	21/26 (81%)
Local patchy shadowing	16/43 (37%)	0/7 (0%)	16/50 (32%)	7/26 (27%)
Bilateral patchy shadowing	9/43 (21%)	0/7 (0%)	9/50 (18%)	5/26 (19%)
Interstitial abnormalities	3/43 (7%)	0/7 (0%)	3/50 (6%)	0/26 (0%)
Pleural fluid	1/43 (2%)	0/7 (0%)	1/50 (2%)	0/26 (0%)
Lymphadenopathy	0/43 (0%)	0/7 (0%)	0/50 (0%)	0/26 (0%)
**Lesion characteristics and location**^b^				
Subpleural	41/43 (95%)	0/7 (0%)	41/50 (82%)	21/26 (81%)
Parallel to the pleura	21/43 (49%)	0/7 (0%)	21/50 (42%)	10/26 (38%)
Vascular thickening shadowing	10/43 (23%)	0/7 (0%)	10/50 (20%)	10/26 (38%)
Upper lobe of the lung	22/43 (51%)	0/7 (0%)	22/50 (44%)	14/26 (54%)
Middle lobe of the lung	9/43 (21%)	0/7 (0%)	9/50 (18%)	6/26 (23%)
Lower lobe of the lung	28/43 (65%)	0/7 (0%)	28/50 (56%)	19/26 (73%)
Posterior segment of lower lung lobes	22/43 (51%)	0/7 (0%)	22/50 (44%)	12/26 (46%)

^a^Data from the first laboratory examination of the patient admission. The normal range of laboratory examination is the standard of *Reference Range Values for Pediatric Care 2nd ed* [31] released by the American Academy of Pediatrics

^b^How many patients have the following lesion location

Of the 26 patients in group C, all had more than two negative consecutive PCR results. However, they all had a history of exposure to COVID-19 infection (or strongly suspected infection), and their chest CT had similar patterns to confirmed patients in group A (Table 2). Fisher exact test results indicated that there was no significant difference in CT characteristics (ground-glass opacity [*p* > 0.05], local patchy shadowing [*p* > 0.05], bilateral patchy shadowing [*p* > 0.05], interstitial abnormalities [*p* > 0.05]) and lesion location (parallel pleura [*p* > 0.05], visible vascular thickening [*p* > 0.05], subpleural [*p* > 0.05], lower lobe of the lung [*p* > 0.05], middle lobe of the lung [*p* > 0.05], upper lobe of the lung [*p* > 0.05]) between groups A and C (Table 3).

**Table 3:** Differences in CT image characteristics between Groups A and C

Characteristics	Group A	Group C	Standardize diff.	***p*** value
**Ground-glass opacity**	–	–	0.31 (− 0.18, 0.80)	0.230
**No**	14 (32.56%)	5 (19.23%)	–	–
**Yes**	29 (67.44%)	21 (80.77%)	–	–
**Local patchy shadowing**	–	–	0.22 (− 0.27, 0.71)	0.380
**No**	27 (62.79%)	19 (73.08%)	–	–
**Yes**	16 (37.21%)	7 (26.92%)	–	–
**Bilateral patchy shadowing**	–	–	0.04 (− 0.44, 0.53)	0.865
**No**	34 (79.07%)	21 (80.77%)	–	–
**Yes**	9 (20.93%)	5 (19.23%)	–	–
**Interstitial abnormalities**	–	–	0.39 (− 0.10, 0.88)	0.168
**No**	40 (93.02%)	26 (100.00%)	–	–
**Yes**	3 (6.98%)	0 (0.00%)	–	–
**Subpleural**	–	–	0.46 (− 0.03, 0.95)	0.095
**No**	2 (4.65%)	5 (19.23%)	–	–
**Yes**	41 (95.35%)	21 (80.77%)	–	–
**Upper lobe of the lung**	–	–	0.05 (− 0.43, 0.54)	0.829
**No**	21 (48.84%)	12 (46.15%)	–	–
**Yes**	22 (51.16%)	14 (53.85%)	–	–
**Middle lobe of the lung**	–	–	0.05 (− 0.44, 0.54)	0.834
**No**	34 (79.07%)	20 (76.92%)	–	–
**Yes**	9 (20.93%)	6 (23.08%)	–	–
**Upper lobe of the lung**	–	–	0.17 (− 0.31, 0.66)	0.492
**No**	15 (34.88%)	7 (26.92%)	–	–
**Yes**	28 (65.12%)	19 (73.08%)	–	–
**Parallel to the pleura**	–	–	0.21 (− 0.28, 0.70)	0.401
**No**	22 (51.16%)	16 (61.54%)	–	–
**Yes**	21 (48.84%)	10 (38.46%)	–	–
**Vascular thickening shadowing**	–	–	0.33 (− 0.16, 0.82)	0.177
**No**	33 (76.74%)	16 (61.54%)	–	–
**Yes**	10 (23.26%)	10 (38.46%)	–	–

Using the Fisher exact test method, *p* < 0.05 was considered to indicate a statistically significant difference

Among the 50 children with positive PCR results, five of them (10%) had negative initial PCR results but showed positive results in subsequent tests. Two of the 50 (4%) had no clinical symptoms and no radiologic findings. Seven of the 50 (14%) were negative for any abnormal CT findings. The spectrum of COVID-19 severity was two (4%) had no symptoms or radiologic signs, five (10%) very mild, 41 (82%) mild, and two (4%) critically ill with one having multiple organ dysfunction syndrome (MODS) and another with renal failure (Table 1). There were no patient mortalities in this study. The critically ill patient with renal failure has since fully recovered.

Among the 43 children with positive PCR results and abnormal CT findings, 41 patients had lesions present in the subpleural area (95%) and lower lung lobes (65%), especially in the posterior segment of the lower lung lobes (22 [78%] of 28). Ground-glass opacities (GGO) were the most common radiologic lesion identified on chest CT (67%). Local patchy shadowing (37%) was the second most common radiologic lesion, followed by local bilateral patchy shadowing (21%, Table 2). Interstitial lesions were rare (7%). Pleural fluid was observed in one case, and no lymphadenopathy was noted (Table 2). Appearances of lesions were irregular shaped, flaky, wedge-shaped, or strip-shaped. The long axis of some lesions (49%) was parallel to the pleura. However, lesions did not follow the segment of the lung lobe, single or multiple, and diffuse consolidation was rare. Bilateral lesions can be seen radiating around the bronchial blood vessels or showing large areas of consolidation, which can be traced by the lung segment into the bronchial tube.

Among the 50 confirmed children (groups A and B), 29 patients (including 23 discharged children) had more than one chest CT. Nineteen of the 29 patients (65%) had improved CT presentations after treatment, and lesions in two of the 19 patients completely disappeared. Two of the 29 patients (7%) showed no change in CT lesions, and 8 of the 29 patients (28%) had more CT lesions after treatment.

**Fig. 2:**
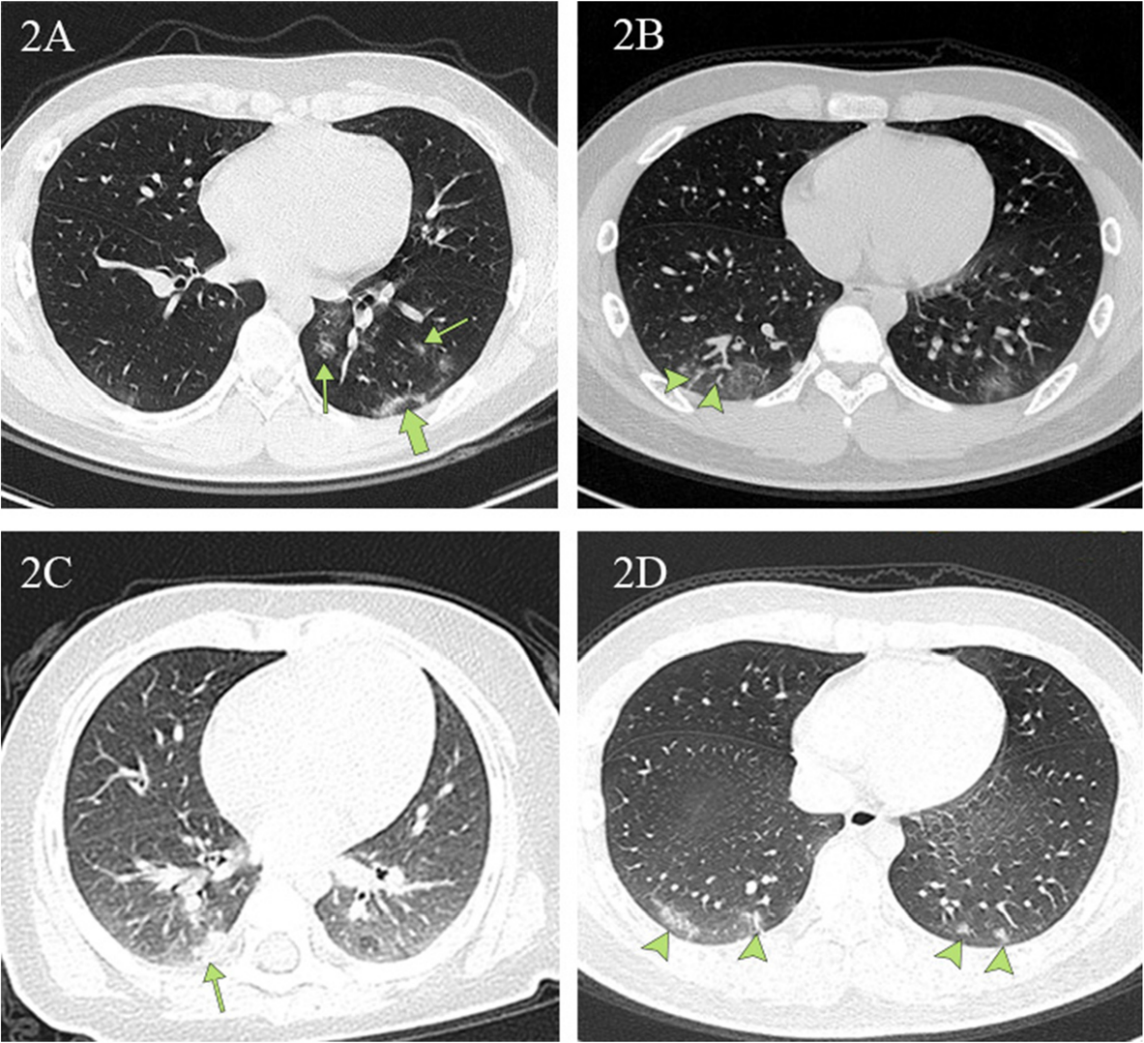
Chest CT images depicting typical radiographic findings of COVID-19 pneumonia in children. **2A** A unilateral chest CT from a 14-year-old boy with a cough. Ground-glass opacities under and parallel to the pleura (thick green arrow) in the inferior lobes of the left lungs. Ground-glass opacities distributed along the bronchovascular bundle (thin green arrow). **2B** Bilateral ground-glass opacities with vascular thickening (arrowheads) in the subpleural area from a 13-year-old boy with a fever and a cough. **2C** Local patchy shadowing (green arrow) image from a 6 month-old girl with a fever and a cough. **2D** Lesions in the lower lobe of both lungs (green arrows) on chest CT obtained from a 15-year-old boy with a fever and a cough

**Fig. 3:**
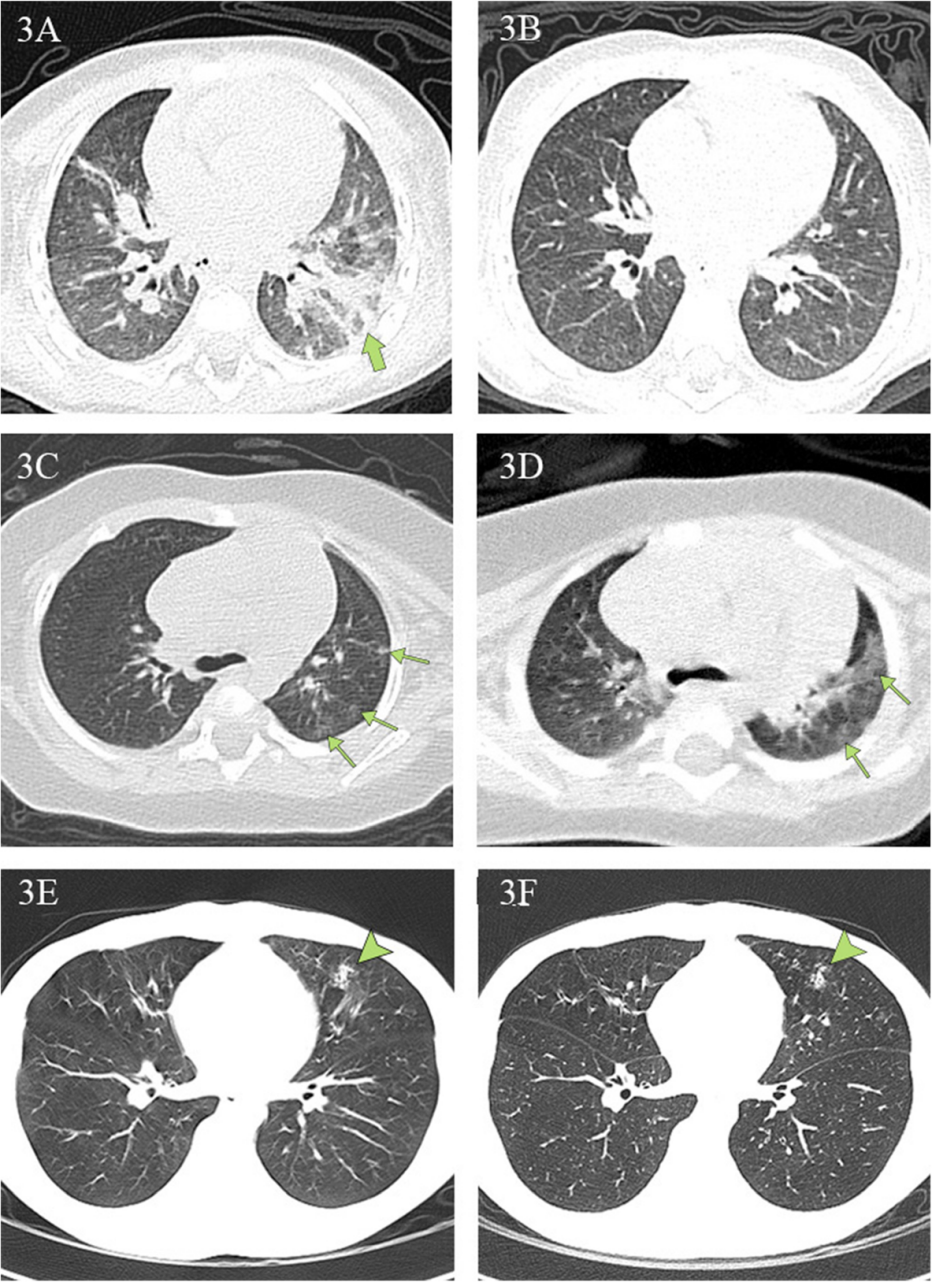
Chest CT findings at initial presentation and at discharge. **3A**, **3B** Chest CT scans obtained from a 1-year-old boy, presenting with fever and diarrhea, at arrival (**3A**) and after (**3B**) treatment. The first CT scan shows a large, patchy shadow in the left inferior lobe (green arrow). The second CT scan shows no lesions. The patient was hospitalized for 17 days prior to discharge. **3C**, **3D** Chest CT scans from a 4-month-old girl, who presented with a fever and a cough at arrival. The first CT scan reveals multiple ground-glass opacities under the pleura in the left superior lobe (green arrows). The second CT scan reveals that the range of original lesions was enlarged and extended to the center. The girl was hospitalized for 13 days and subsequently discharged. **3E**, **3F** Chest CT scans from a 14-year-old boy, presenting with rhinorrhea and a cough, at arrival and discharge. The first CT scan reveals a patchy shadow in the left middle lobe (arrowhead). There were no obvious changes in the areas of pulmonary consolidation on the second CT scan. The boy was hospitalized for 11 days and then discharged

Figure 2 illustrates typical radiographic features of COVID-19 pneumonia in children. Figure 3 shows chest CT before and after treatment from three COVID-19 children. Cox regression results (Table 4) indicated that there is no association between changes in CT lesions (completely absorbed [*p* > 0.05], partially absorbed [*p* > 0.05], worse [*p* > 0.05]). Table 5 lists changes in CT lesions during treatment. Table 6 lists the normal ranges for children of different ages based on *Reference Range Values for Pediatric Care 2nd edition* pages 92–98 [31].

**Table 4:** Association between CT imaging changes and clinical outcome^a^

Exposure	Adjust^b^
**Changes in CT images during treatment**	
**Completely absorbed**	1.0
**Partially absorbed**	0.56 (0.25, 1.28), 0.168
**No change**	0.11 (0.01, Inf)^c^
**Worse**	0.34 (0.10, 1.13), 0.08

^a^Using the time-vary Cox regression method, *p* < 0.05 was considered to indicate statistically significant difference

^b^Adjusted for gender, age, PCR positive and CT positive

^c^This model failed when analyzing “no change in CT image” due to the small sample

**Table 5:** Changes in CT presentation after treatment

Result	Completely absorbed	Partially absorbed	No change	Worse
**Between the first and discharge CT**^a^	1/15 (7%)	10/15 (67%)	2/15 (13%)	2/15 (13%)
**Between the first and the nearest CT**^b^	1/8 (13%)	4/8 (50%)	0/8 (0%)	3/8 (37%)
**Between the first and latest CT**^c^	2/29 (7%)	17/29 (58%)	2/29 (7%)	8/29 (28%)

^a^Only for those discharged patients with at least two CT; discharge CT here means CT within 2 days of discharging; total 15 patients out of group A and group B

^b^Only for those discharged patients with at least two CT but no CT within 2 days of discharging; the nearest CT means that the CT taken closest to the date of discharging; total 8 patients out of group A and group B

^c^For all patients with at least two CT; total 29 patients out of group A and group B

**Table 6:** Laboratory examination reference ranges

Age	Sex	Hemoglobin (g/dL)	C-reactive protein (mg/L)	Platelets (× 10^**9**^/L)	Lymphocytes (× 10^**9**^/L)	Leukocytes (× 10^**9**^/L)
**1 to < 2 months**	M	102–127	0.09–10.41	221–471	2.22–5.63	8.36–13.66
	F	111–137		184–430	2.49–6.26	7.34–12.32
**2 to < 6 months**	M	105–130		215–448	2.57–7.54	7.91–13.41
	F	107–134		147–423	2.22–7.11	6.85–12.84
**6 months to 2 years**	M	104–125		185–399	2.47–6.41	7.73–13.12
	F	108–126		211–408	2.34–6.44	7.05–12.98
**3–5 years**	M	114–143		187–444	1.60–5.30	4.40–12.9
	F	114–143		187–444	1.60–5.30	4.40–12.9
**6–8 years**	M	115–143		186–400	1.40–3.90	3.80–10.4
	F	115–143		186–400	1.40–3.90	3.80–10.4
**9–10 years**	M	118–147		186–400	1.40–3.90	3.80–10.4
	F	118–147		186–400	1.40–3.90	3.80–10.4
**11–14 years**	M	124–157		176–381	1.00–3.20	3.80–10.4
	F	119–148		176–381	1.00–3.20	3.80–10.4
**15–19 years**	M	133–169		138–319	1.00–3.20	3.80–10.4
	F	119–148		158–361	1.00–3.20	3.80–10.4

From *Reference Range Values for Pediatric Care 2nd edition* pages 92–98 [31] released by the American Academy of Pediatrics

## Discussion

The symptoms in children with COVID-19 infection have been well described in the literature [9–18]. Our results are consistent with these previous reports. For example, the clinical symptoms from our study versus the recent study with the most pediatric patients are similar [10]: fever, 64% versus 41.5%; cough, 44% versus 48.5%; diarrhea, 6% versus 8.8%; and fatigue, 4% versus 7.6%. Results including ours indicate that COVID-19 symptoms in children follow a similar pattern in adults, albeit much less severe.

Our results of abnormal laboratory findings for children infected with COVID-19 contrast with recently published ones [15, 22–24]. For example, our results for lymphopenia compared to Zheng et al. are 16% versus 40% [22]. Their normal reference values for lymphocytes were (2.1–5.7) ×  10^9^/L (< 3 years), (1.4–4.2) × 10^9^/L (4–6 years), and (1.1–3.2) × 10^9^/L (≥ 6 years). To date, this is the only paper that has explicitly listed the normal ranges for children of different age ranges [22]. Thus, the differences between ours and those in the literature are most likely due to different normal ranges used for children of different ages or the small number of children who participated in their studies.

Like clinical symptoms, the laboratory findings in COVID-19-positive pediatric patients can vary from adult patients. Guan et al. [25] noted that 731 (82%) of 890 adult patients had lymphopenia, whereas only eight (16%) children had lymphopenia in this study. Similarly, 481 (61%) of 793 adult patients were found to have an elevated C-reactive protein. In contrast, only ten (20%) children in this study had elevated C-reactive protein. Some laboratory findings were consistent between children and adult groups: leukopenia 38% versus 36% and thrombocytopenia 14% versus 18%. The mechanism behind the observations is unknown and might provide an explanation for the differences between pediatric and adult patients.

The most common pattern of chest CT is ground-glass opacities, followed by local patchy shadowing and then local bilateral patchy shadowing, which is consistent with published data [9–18]. Our study indicates that chest CT manifested with a predominance of lesions in the subpleural area (41 [95%] of 43) and in lower lung lobes (28 [65%] of 43), especially in the posterior segment (22 [78%] of 28), an area with a relatively dense amount of bronchioles, blood vessels, and alveoli. To the best of our knowledge, these are the first quantitative results on the locations of chest CT lesions for COVID-19 children [21]. COVID-19 is less severe in children than in adults, and the children infected with COVID-19 were at the early stages of the disease when admitted to the hospital. The fact that an overwhelming percentage of pediatric patients had lesions in the subpleural area suggests this site is the first target for the COVID-19 virus.

The current gold standard for the diagnosis of COVID-19 is PCR. However, it has been documented that patients with a negative PCR result cannot be definitively ruled out for COVID-19 infection [11, 26]. Our results are consistent with the literature. Among the 50 hospitalized children with positive PCR results, five of them (10%) had negative initial PCR results but showed positive results in subsequent tests. Moreover, 26 patients in group C never had a positive PCR result but had histories of contact with COVID-19 patients. Most of them exhibited clinical symptoms such as fever (81%) and cough (73%). Although they received a negative PCR result at least twice, all 26 patients had similar CT patterns to the PCR-positive COVID-19 patients in group A. Twenty-one (81%) had ground-glass opacities (GGO). Seven (27%) had local patchy shadowing. Five (19%) had bilateral patchy shadowing. Furthermore, our Fisher exact analysis indicated that there was no significant difference in CT image characteristics and lesion location between groups A and C. Although a positive CT alone cannot rule out the possibility of other causes of virus-induced pneumonia [11, 26], all 26 children were hospitalized and given immediate antiviral and supportive therapy. Whether or not a child presents with pneumonia is one of the key considerations for clinical management, and it is crucial to start treatment as early as possible, considering that many deaths in the adult population are due to complications resulting from severe pneumonia [3–7].

It has been well documented that chest CT is a powerful tool to identify and characterize pneumonia for COVID-19 adult patients [25–28]. However, there is no publication to study its usefulness in evaluating clinical recovery for children with COVID-19 infection. To determine whether CT is necessary, we investigated the data of 23 patients who had been discharged after effective treatment and had at least two CT scans. All patients had normal body temperatures for more than 3 days at the time of discharge, clinical symptoms disappeared, and PCR tests all returned negative twice at 24-h intervals. Of the 23 children, eight patients did not receive CT scans within the 2 days before their discharge. However, in their most recent CT scan performed in the hospital, most children either still had lesions (50%), or more developed lesions since the previous scan (37%). The remaining 15 discharged children had a CT obtained within 2 days of discharge. Again, ten patients had lesions that were not completely absorbed (67%), two were the same (13%), and lesions in another two became worse (13%). These results indicate that CT may not be better than symptoms in evaluating recovery. Our Cox regression analysis further showed that there was no association between changes in CT lesions and clinical outcomes. The results are consistent with the knowledge that clinical improvement predates radiographic improvement by weeks for children with community-acquired pneumonia.

When deciding whether to use CT on children, the harmful effects that radiation may have on a growing body must be considered. Hong et al., in a study of 12,068,821 children aged 0 to 19 years, found a statistically significant increase in cancer in children exposed at least once to diagnostic low-dose ionizing radiation after adjusting for age and sex [29, 30]. Based on our data, we do not recommend using CT for determining clinical recovery unless it is necessary to evaluate the status of pneumonia. For comparison, the current criteria for discharging adult patients infected with COVID-19 in China are (1) normal body temperature for 3 days, (2) two negative PCR tests at 24-h intervals, (3) resolution of clinical symptoms (these three are the current criteria for discharging pediatric patients in this hospital), plus (4) a chest imaging requirement: pulmonary imaging must show significant absorption of lesions. To date, there are no child-specific discharge criteria for COVID-19 in China.

Our study had a few limitations. First, this study has a small sample size and was conducted at a single-center in Wuhan, China, located at the center of the outbreak. The clinical severity of pediatric patients outside Wuhan may be less severe. Indeed, it is reported that there is a lower death rate of adult patients outside Wuhan areas. Second, long-term follow-up was not done because of the short time for data collection.

## Conclusions

The severity of COVID-19 infection in children is less than it is in adults in terms of symptoms, lung consolidation as visualized by CT, and laboratory abnormalities. COVID-19 has a preference for subpleural areas of the lung in pediatric patients. Chest CT is an excellent tool to detect and characterize COVID-19 pneumonia but not to evaluate the resolution of illness for children.

## Data Availability

The datasets used and/or analyzed during the current study are available from the corresponding author on reasonable request.

## Abbreviations

BPS: Bilateral patchy shadowing
COVID-19: Coronavirus disease 2019
CT: Computerized tomography
GGO: Ground-glass opacities
LPS: Local bilateral shadowing
MERS: Middle Eastern respiratory syndrome
MODS: Multiple organ dysfunction syndrome
PCR: Polymerase chain reaction
SARS: Severe acute respiratory syndrome
